# Investigating Performance of cVEMP and oVEMP in the Identification of Superior Canal Dehiscence in Relation to Dehiscence Location and Size

**DOI:** 10.3390/audiolres11030042

**Published:** 2021-09-09

**Authors:** Maxime Maheu, Ahlem Elblidi, Issam Saliba

**Affiliations:** 1 Faculty of Medicine, School of Speech Language Pathology and Audiology, University of Montreal, Montreal, QC H3N 1X7, Canada; 2 Centre de Recherche Interdisciplinaire en Réadaptation, Institut Universitaire sur la Réadaptation en Déficience Physique de Montréal (IURDPM), Pavillon Laurier, CIUSSS du Centre-Sud-de-l’Île-de-Montréal, Montreal, QC H2H 1C4, Canada; 3 Montreal University Hospital Center (CHUM), Montreal, QC H2X 3E4, Canada; ahlem.elblidi@gmail.com; 4 Department of Surgery, Division of Otorhinolaryngology-Head & Neck Surgery, University of Montreal, Montreal, QC H3C 3J7, Canada

**Keywords:** superior semicircular canal dehiscence, vestibular evoked myogenic potentials, vestibular

## Abstract

Compare the sensitivity and specificity of cVEMP (500 Hz), oVEMP (500 Hz and 4 kHz) in the identification of SSCD. A secondary objective was to identify the influence of dehiscence size and location on cVEMP and oVEMP responses. **Methods**: Individuals with unilateral (n = 16) and bilateral (n = 10) scan confirmed SSCD were assessed using air-conducted cVEMP and oVEMP **Results**: For cVEMP, an amplitude cutoff of 286.9 μV or a threshold cutoff of 67.5 dBnHL revealed, respectively, a sensitivity of 75% and 70.6% and a specificity of 69.4% and 100%. For oVEMP (500 Hz), an amplitude cutoff of 10.8 μV or a threshold cutoff of 77.5 dBnHL revealed a sensitivity of 83.33% and a specificity of 87.5% and 80%, respectively. oVEMP (4 kHz), an amplitude cutoff of 3.1 μV, revealed a high specificity of 100% but a low sensitivity of 47.2%. A positive correlation was noted between the length of the SSCD and the cVEMP and oVEMP (500 Hz) thresholds and cVEMP amplitude. **Conclusions**: Our results support the use of oVEMP in the identification of SSCD. The presence of oVEMP (500 Hz) with an amplitude higher or equal to 10.8 μV, a threshold lower or equal to 77.5 dBnHL or oVEMP (4 kHz) amplitude of 3.1 μV represents the most useful to identify SSCD.

## 1. Introduction

The peripheral vestibular system, located in the inner ear, comprises three semicircular canals (superior, horizontal and posterior) and two otolithic organs (utricle and saccule), which, respectively, detect angular and linear accelerations of the head [1]. It has been demonstrated that otolithic organs could be stimulated using sound and vibration [2]. This sensitivity to sound and vibration has led to the development of a well-documented clinical test, the vestibular evoked myogenic potential (VEMP). Two subtypes of VEMP responses are most commonly used: the cervical vestibular evoked myogenic potential (cVEMP) and the ocular vestibular evoked myogenic potential (oVEMP). The cVEMP is an inhibitory response measured at the level of the ipsilateral sternocleidomastoid muscle assessing the function of the sacculo-colic pathway [3]. The oVEMP is an excitatory response measured at the level of the contralateral inferior oblique muscle, which measures the function of the utriculo-ocular pathway [4]. Giving its widespread accessibility in clinical and research settings, there was a growing interest in cVEMP and oVEMP use in the identification of multiple vestibular diseases [5].

Of particular interest, investigation of the superior semicircular canal dehiscence (SSCD) using VEMP responses showed promising results, giving its pathophysiology. Indeed, SSCD is an inner ear pathology involving the thinning or the dehiscence of the bone at the level of the superior semicircular canal, creating a third mobile window, which lowers endolymph impedance allowing the stimulation of the peripheral vestibular system following acoustic stimulation [6]. These patients report phonophobia, autophony, pulsatile tinnitus, and imbalance [7]. Therefore, even though the gold standard in the identification of SSCD is still the visualization of the dehiscence using high-resolution CT-scan (HRCT-scan), VEMPs have been demonstrated to show larger amplitudes and reduced thresholds [6].

cVEMP capability of separating healthy from SSCD ears was assessed and showed variable results [8,9,10,11]. These studies proposed to use the threshold value as a cutoff to suggest the presence or absence of SSCD, which results in great variability for sensitivity (57–100%) and specificity (69–100%) as a function of the chosen cutoff threshold. Other authors assessed the usefulness of the oVEMP in the identification of SSCD following a 500 Hz tone burst [10,11,12,13] or following a high-frequency tone burst [13,14]. The sensitivity and specificity for oVEMP (500 Hz) in the identification of SSCD vary between 62% to 100% and 73% to 100%, respectively. Again, these different studies used different amplitude cutoff criteria, which may explain the large variability in the results [10,11,12]. Instead of a cutoff based on amplitude, other researchers used an amplitude asymmetry ratio above 40% as a cutoff [13]. More recently, high-frequency VEMP stimulus has been proposed to be highly sensitive to identify SSCD ears [14,15]. These authors suggested that the presence of oVEMP evoked response following a 4 kHz stimulation could distinguish between healthy and dehiscent ears (sensitivity: 100%; specificity: 100%). However, this high sensitivity and specificity were not replicated in a recent study where the authors observed a sensitivity of 83% and a specificity of 93% in a population of participants reporting dizziness [13].

As described, the variable methodology used in these previous studies reduces the ability to compare the results and may contribute to the great variability in the sensitivity and specificity of VEMP responses. Moreover, as it has been previously reported, the location of the dehiscence may influence the cVEMP results [15]. This may as well explain, at least in part, the variability in VEMP parameters between studies, but most of the previous reports did not account for the location of the dehiscence. Finally, this relation between VEMP parameters and dehiscence location has never been explored using oVEMP. Therefore, the main objective of this study was to compare the sensitivity and specificity of the tone-burst cVEMP (500 Hz), oVEMP (500 Hz and 4 kHz) in the identification of SSCD in a clinical population of individuals with unilateral and bilateral scan confirmed SSCD. A secondary objective was to identify the influence of dehiscence parameters (size and location) on cVEMP and oVEMP parameters (amplitude and threshold).

## 2. Methodology

### 2.1. Participants

**Figure 1:**
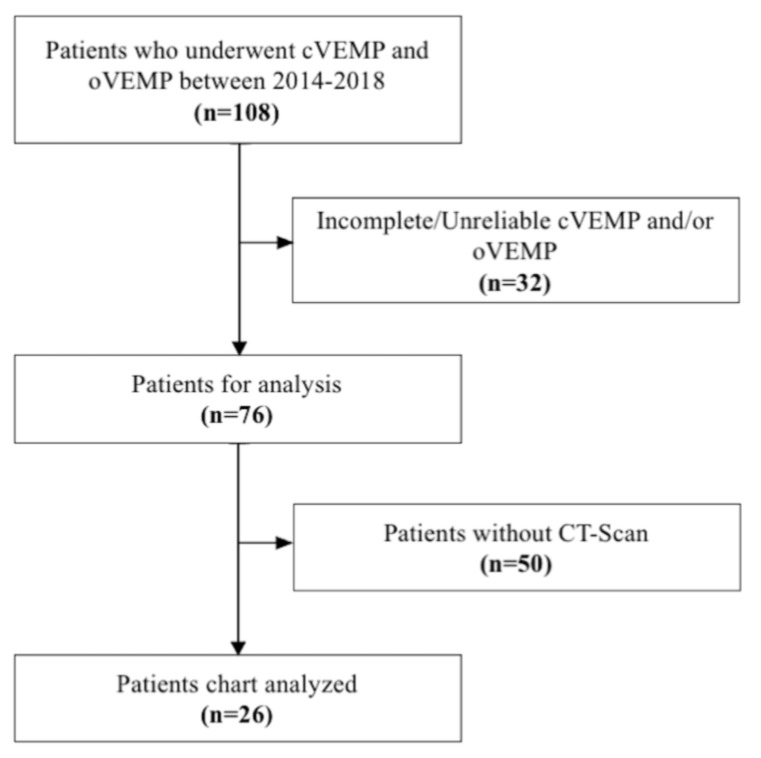
Flowchart representing the selection of the participants from the original database.

A retrospective chart study was performed on 108 consecutive adults complaining of dizziness who underwent cVEMP and oVEMP testing between 2015 and 2018. Based on our previously published selection protocol [16] (Benamira et al., 2014), a total of 36 SSCD ears were selected for surgery and received surgical confirmation (10 patients with bilateral SSCD and 16 patients with unilateral SSCD) and were included in this study. Patients with incomplete data or with near dehiscence were excluded. The patients were aged between 30 and 78 years (mean: 54.33 years old ±12.78). The contralateral ear of the 16 patients with unilateral SSCD was used as a control group (non-SSCD) to determine the sensitivity and specificity of the VEMP in identifying SSCD ears. No significant difference between groups was seen for age (non-SSCD ears: mean age = 54.29 years old ±12.38; SSCD ears: mean age = 54.13 years old ±13.44; *p* = 0.968). Figure 1 shows the inclusion/exclusion flowchart of patients. The study was approved by our institutional research ethics board and followed the standards of our institutional ethics committee.

### 2.2. Protocol

Each patient underwent cVEMP and oVEMP testing in the same session. Usually, cVEMP testing was performed first to limit the effect of fatigue. For both cVEMP (500 Hz) and oVEMP (500 Hz and 4 kHz), we analyzed the peak-peak amplitude of the response at 95 dBnHL (125 dBpeSPL) and the threshold (only for 500 Hz tone-burst stimulus). A response was considered present when the first component (P1 for cVEMP and N1 for oVEMP) was clearly observed and replicable.

### 2.3. Vestibular Evoked Myogenic Potentials (cVEMP and oVEMP)

The VEMP method used is based on the recent recommendations [5] using NavPro Bio-logic (Natus, Germany). The stimulation used was an air-conducted 95 dBnHL tone burst (cVEMP: 500 Hz; oVEMP: 500 Hz and 4 kHz) with a rise/fall time of 1 ms, a plateau of 2 ms, a stimulation rate of 5.1/s and 120 sweeps.

For the cVEMP, the responses were recorded using surface electrodes placed on the mid forehead (ground), on the belly of the ipsilateral sternocleidomastoid muscle (active) and on the upper sternum (reference). The equipment used did not allow to measure for EMG contraction level. However, we used a well-demonstrated technique to reduce EMG variability [17]. Indeed, for each cVEMP trial, participants were lying supine in a reclined position (bed angle 30 degrees) and were required to lift their head and turn it away from the stimulated ear in order to elicit appropriate and replicable contraction level of the SCM [5,18].

On the other hand, the oVEMP responses were measured using surface electrodes placed just beneath the midpoint of the eye contralateral to the stimulated ear (active), another electrode 1 cm beneath the active (reference) and the ground on the mid forehead. During oVEMP recording, patients were seated with the head kept in a straight position, and gaze was elevated at maximal comfortable up-gaze position [18].

### 2.4. High-Resolution CT-Scan

**Figure 2:**
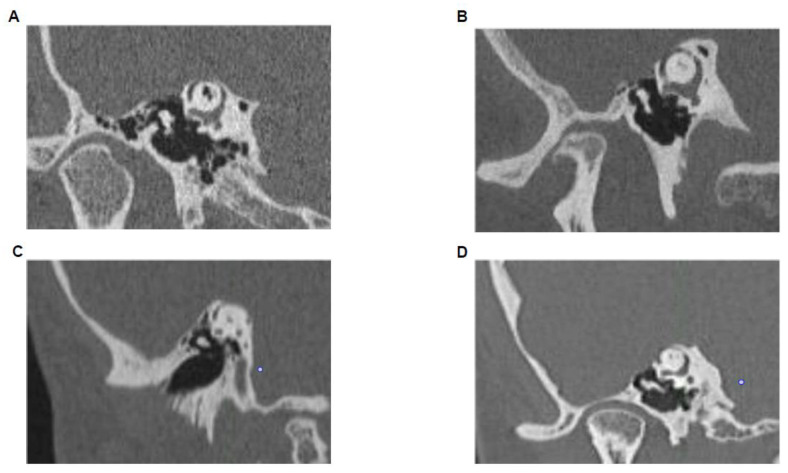
CT-Scan image representing each location. (**A**) Superior; (**B**) anterior; (**C**) posterior superior; (**D**) anterior superior.

The data from high-resolution thin-section (0.6 mm) multi-detector row CT scans of the temporal bone from patients who underwent surgery for SSCD were retrospectively reviewed. For each ear, reformatted images were created in the coronal plane and in the plane of the superior semicircular canal (i.e., Poeschl view) [19]. On each plane, we assigned a value of “dehiscent”, “not dehiscent” or “near dehiscent”. Near dehiscent is defined as a thin bone overlying the superior semicircular canal rather than frank dehiscence to the bony covering [20]. In this study, near-dehiscent ears were excluded. The size of the dehiscence was calculated based on the length between its distinct ends on the Poeschl plane [21]. The location of the dehiscence was also described to be anterior (on the anterior foot of the canal near the ampulla), superior (corresponding to the dome of the canal) or posterior (on the posterior foot of the canal) (Figure 2) [22]. All CT-scan images were read by a neurotologist and confirm by another one; both of them were unaware of the VEMP results. Since it is a retrospective study, we were not able to report data of the intra-operative superior canal dehiscence measurement, and we excluded some operated patients because of their incomplete data.

### 2.5. Analysis

First, we analyzed the difference in amplitude and threshold between groups (non-SSCD and SSCD) using an ANOVA of two groups (non-SSCD; SSCD) by two response parameters (peak-peak amplitude and threshold). One ANOVA was performed using cVEMP data, and another ANOVA was used to assess oVEMP (500 Hz) data. Bonferroni correction factor was applied when necessary.

Second, receiver operating characteristics (ROC) curves were also analyzed for each VEMP parameter to determine the sensitivity, specificity and associated cutoff criteria.

Thirdly, an ANOVA 2 groups (anterior superior; superior) by 2 responses parameters (peak-peak amplitude and threshold) was performed to assess any effect of SSCD location on VEMP parameters. One ANOVA was performed using cVEMP data, and another ANOVA was used to assess oVEMP (500 Hz) data. Table 1 describes the different VEMP results for each group of dehiscence locations. For analysis, we compared only the anterior-superior (n = 15) and superior (n = 17) locations because the small sample size of the other groups prevents them from performing appropriate statistical.

Finally, bivariate correlations were performed between various VEMP parameters and SSCD size with a specific focus on SSCD location. Analysis and figures were performed using Matlab (R2020a).

## 3. Results

### 3.1. cVEMP

**Figure 3:**
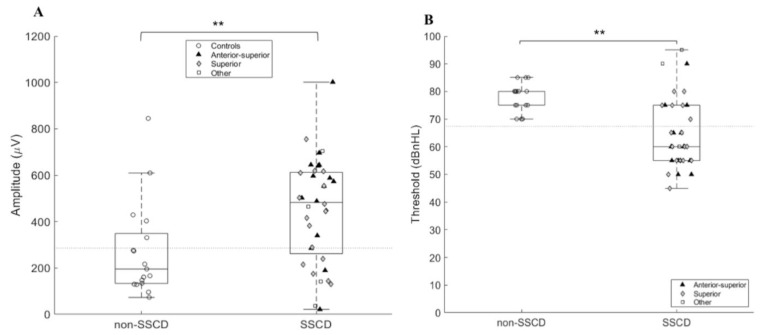
Boxplot and scatter of cVEMP amplitude (**A**) and cVEMP threshold (**B**) for both groups. The dotted line represents the proposed cutoff. ** *p* ≤ 0.001.

As expected, the uncorrected peak-peak amplitude of the cVEMP response was significantly larger (*p* = 0.008) in the SSCD ears (447.97 μV ± 224.22) as opposed to the non-SSCD ears (271.64 μV ± 203.93) (Figure 3A). Moreover, the cVEMP thresholds were significantly lower (*p* ≤ 0.0001) in the SSCD ears (65.25 dBnHL ± 11.99) as opposed to the non-SSCD ears (78.13 dBnHL ± 5.13) (Figure 3B).

**Figure 4:**
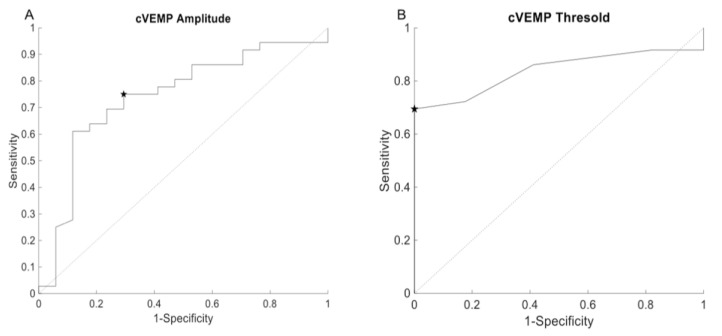
ROC curve representing the diagnostic value of cVEMP amplitude (**A**) and cVEMP threshold (**B**). The star symbol represents sensitivity and specificity for the given cutoff value.

The ROC-curve analysis revealed an area under the curve of 0.74 for cVEMP amplitude and 0.84 for cVEMP threshold. An amplitude cutoff of 286.9 μV revealed a sensitivity of 72.22% and a specificity of 70.6% (Figure 4A). Using a threshold cutoff of 67.5 dBnHL revealed a sensitivity and specificity of 69.4% and 100%, respectively (Figure 4B).

We can observe that location of the dehiscence influence cVEMP parameters. No significant differences between locations (anterior-superior and superior) for cVEMP amplitude (*p* = 0.065) nor cVEMP threshold (*p* = 0.691) were found.

### 3.2. oVEMP (500 Hz)

**Figure 5:**
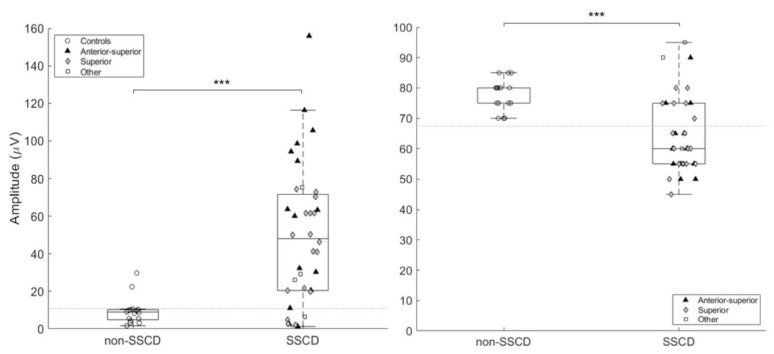
Boxplot and scatter of oVEMP amplitude (**A**) and oVEMP threshold (**B**) for both groups. The dotted line represents the proposed cutoff. *** *p* ≤ 0.0001.

The results for the oVEMP (500 Hz) using peak-peak amplitude or threshold differed significantly between the two groups. Indeed, peak-peak amplitude was significantly larger (*p* ≤ 0.0001) in SSCD group (49.56 μV ± 36.36) as opposed to the non-SSCD group (9.49 μV ± 7.22) (Figure 5A) and thresholds were significantly lower (*p* < 0.0001) for the SSCD group (64.58 dBnHL ± 13.22) as opposed to the non-SSCD group (82.33 dBnHL ± 5.93) (Figure 5B).

**Figure 6:**
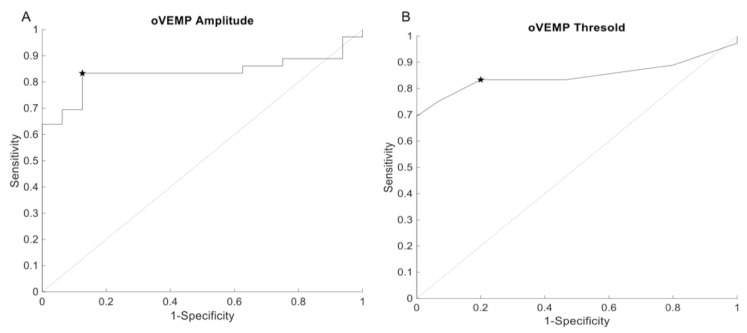
ROC curve representing the diagnostic value of oVEMP (500 Hz) amplitude (**A**) and oVEMP (500 Hz) threshold (**B**). The star symbol represents sensitivity and specificity for the given cutoff value.

The ROC-curve analysis revealed an area under the curve of 0.84 for oVEMP peak-peak amplitude and 0.85 for oVEMP threshold. Using an amplitude cutoff of 10.8 μV revealed a sensitivity and specificity of 83.33% and 87.5%, respectively (Figure 6A). For the oVEMP threshold criteria, a cutoff of 77.5 dBnHL revealed a sensitivity and specificity of 83.33% and 80%. Lowering the threshold cutoff to 67.5 dBnHL increase the specificity to 100% but reduced sensitivity to 69.4% (Figure 6B).

No significant difference was found between locations (anterior-superior and superior) for oVEMP amplitude (*p* = 0.08) nor oVEMP threshold (*p* = 0.157).

### 3.3. oVEMP (4 kHz)

**Figure 7:**
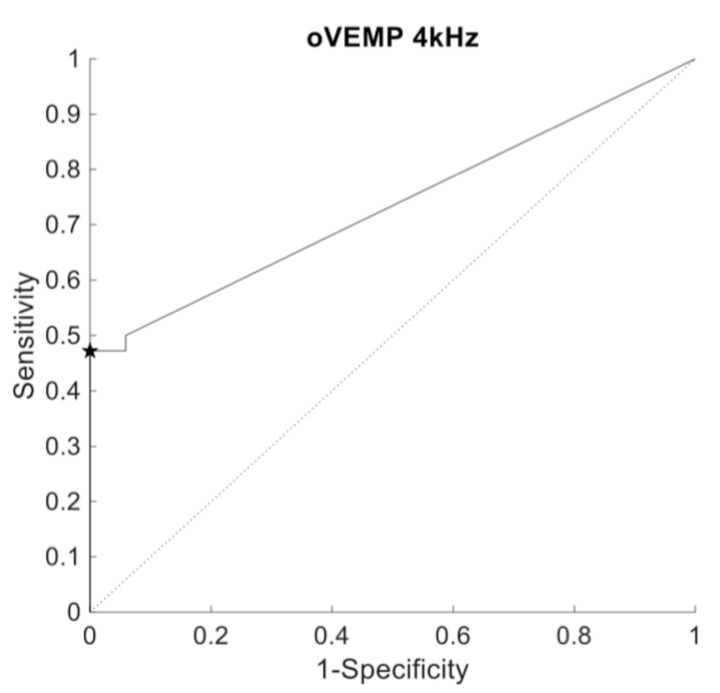
ROC curve representing the diagnostic value of oVEMP (4 kHz) amplitude. The star symbol represents sensitivity and specificity for the given cutoff value.

Response of the oVEMP (4 kHz) was present in 5.9% (n = 1) and absent in 94.1% (n = 16) of the ears without positive HRCT-scan (n = 17). In the group of confirmed SSCD ears (n = 42), oVEMP (4 kHz) response was identified in 50% (n = 21) and absent in 50% (n = 21). The ROC-curve analysis revealed an area under the curve of 0.73 for oVEMP 4 kHz peak-peak amplitude. Using an amplitude cutoff of 3.1 μV revealed a sensitivity and specificity of 47.2% and 100%, respectively (Figure 7). Because of the small sample of non-SCCD ears with an evoked oVEMP at 4 kHz, no statistics could be performed to compare amplitude differences between groups.

For the ears with SSCD in the anterior-superior location, the oVEMP (4 kHz) was present in 66.7% (n = 10) and absent in 33.3% (n = 5) of the ears. On the other hand, for the ears with SSCD in superior location, the oVEMP (4 kHz) was present in 29.4% (n = 5) and absent in 70.6% (n = 12). The relation between SSCD location and presence of oVEMP (4 kHz) is significant (Khi-Square = 4.44, ddl = 1, *p* = 0.035) suggesting a higher sensitivity of oVEMP (4 kHz) for dehiscence located in the anterior-superior region, which is closer to the ampulla.

### 3.4. SSCD Size and VEMP Results

**Figure 8:**
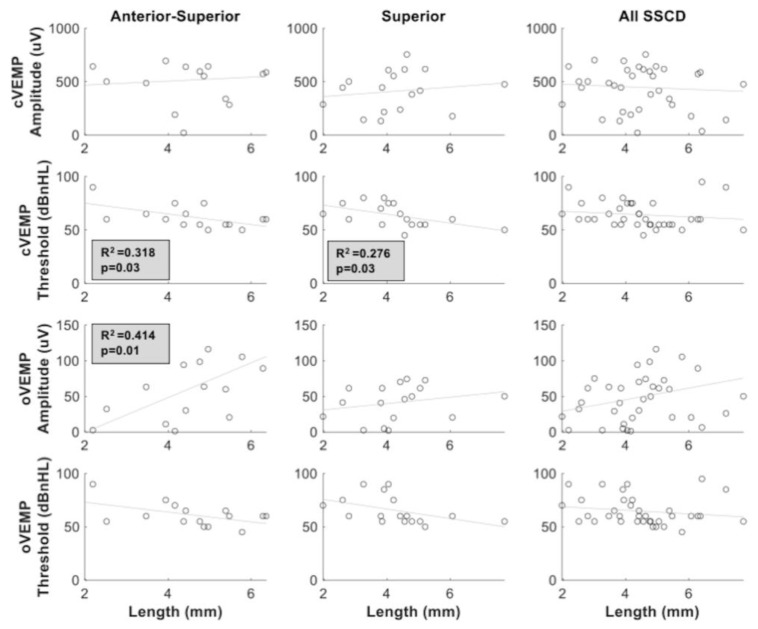
Correlations between cVEMP and oVEMP parameters (amplitude and threshold) and length of dehiscence for each location group anterior-superior, superior and all locations (anterior-superior, superior, posterior superior, anterior). Significant correlations between length of dehiscence and cVEMP thresholds are observed within the anterior-superior and superior group and between the length of dehiscence and oVEMP amplitude within the anterior-superior group. Shaded plot area represents significant correlations (*p* < 0.05).

Figure 8 compares the relation between the length of dehiscence and VEMP parameters for the anterior-superior group, the superior group and all SSCD locations merged together. A significant relation was observed between length of dehiscence and cVEMP threshold for the anterior-superior (*p* = 0.03; R^2^ = 0.318) and superior group (*p* = 0.03; R^2^ = 0.276), but not when all SSCD locations were merged. Moreover, the length of dehiscence was significantly correlated with oVEMP amplitude only in the anterior-superior group (*p* = 0.01; R^2^ = 0.414). No significant relation between the length of dehiscence and VEMP parameters was observed in the superior group.

## 4. Discussion

The present study had for primary objective to assess the sensitivity and specificity of the cVEMP and oVEMP parameters in the identification of SSCD. The results support the use of the oVEMP (500 Hz) threshold and amplitude in the identification of SSCD as they revealed the highest sensitivity and specificity using a cutoff of 77.5 dBnHL and 10.8 μV, respectively. This is in line with previous findings suggesting high sensitivity and specificity of tone-burst oVEMP in the identification of SSCD [10,11,12,23]. The sensitivity and specificity found in our study are similar to those of Hunter et al. [11] but less as compared to the others [10,12,24]. Even though the stimulation parameters between these studies and the present manuscript are the same, one slight difference is present in the electrode montage. Indeed, they used a montage where the reference electrode was 2 cm below the active and where the ground is placed on the sternum. In our study and in Hunter et al.’s [11] study, the ground electrode was placed on the forehead. The effect of ground and reference electrode position may have influenced the quality of the recording [25]. Moreover, differences in stimulus used may reduce comparability between the studies as some used click and others used tone burst of different frequencies.

For the high-frequency oVEMP (4 kHz), the present study shows similar high specificity but contrasts with a greatly reduced sensitivity as opposed to previous studies [13,14]. Our results suggest a sensitivity of 50% as opposed to 100% for previous reports. The lack of sensitivity in our study may be in line with the stimulus used. Indeed, we used a less intense stimulus (95 dBnHL) as opposed to Lin et al. [13] (97 dBnHL). Moreover, our rise/fall and plateau duration differed significantly from these studies, reducing the total duration of our tone burst. This has been shown to significantly reduce amplitude response as it reduces the energy level delivered to the ear [5]. Therefore, this might have led to a lower response rate, reducing the sensitivity of the test. Further studies should therefore investigate this hypothesis. In addition, the location of the SSCD seemed to greatly influence the presence or absence of oVEMP evoked response at 4 kHz. Indeed, the group with the dehiscence closer to the ampulla (anterior-superior) showed a significantly higher response rate. Therefore, differences in the distribution of the dehiscence location could explain, at least in part, the lack of sensitivity of oVEMP (4 kHz) in the identification of SSCD.

On the other hand, the cVEMP amplitude and threshold criteria revealed to be less sensitive and less specific in identifying the involved ear and therefore should be interpreted with caution. This is in line with previous reports suggesting lower sensitivity and specificity of the cVEMP parameters [8,9,10,11]. It has been proposed that the lower sensitivity and specificity of cVEMP in the identification of SSCD could be related to the inhibitory nature of the evoked potential [3]. Indeed, it was observed that cVEMP amplitudes tend to saturate in SSCD ears at higher intensities as opposed to oVEMP amplitudes [2]. Therefore, it is possible to hypothesize that the lack of sensitivity and specificity using cVEMP amplitude may also be in line with the influence of EMG level on cVEMP amplitude, but this could not be observed as we did not monitor EMG. Future studies should investigate this hypothesis.

Our results confirm a positive relation between cVEMP threshold, oVEMP amplitude, oVEMP threshold and length of dehiscence as measured by HRCT-scan, which is in line with previous findings [11] but only for some specific location of the dehiscence. In addition, these results are similar to our previous study reported by Saliba et al. [7], where no significant correlations were measured between VEMP parameters and length of dehiscence when all SSCD locations are merged together. As our results suggest, the location of the dehiscence has a major influence on the association between length and VEMP parameters. Indeed, we measured significant correlations only within groups where SSCD was closer to the ampulla (anterior-superior and superior), but the strength of the association was always stronger for the anterior-superior group. The influence of dehiscence location on VEMP results is supported by previous literature [26], where they observed that the closer the dehiscence was to the ampulla, the lower the cVEMP threshold. The influence of dehiscence location on VEMP results is supported by previous literature [27], where they observed that the greatest displacement occurs at the location of the dehiscence. Therefore, if the dehiscence is closer to the ampulla, one could hypothesize a lower VEMP threshold and larger VEMP amplitude. Therefore, the location of the dehiscence should be considered in future studies as this could be explaining the differences between the studies.

Based on the results of the present study and on previous literature, we suggest that cVEMP testing is not required in the evaluation of SSCD as it lacks sensitivity and specificity. However, we strongly recommend the use of oVEMP (500 Hz) with an amplitude cutoff of 10.8 μV and threshold cutoff of 77.5 dBnHL as it reveals to have great sensitivity and specificity in the identification of SSCD. The presence of oVEMP evoked response at 4 kHz is highly specific to SSCD and should be included in the investigation of SSCD. However, further studies to determine optimal stimulus parameters in order to increase its sensitivity.

Limitation of the study: Moreover, given the specific aim of the present study, which aimed specifically at the use of VEMP in the identification of SSCD, we did not assess the combination of multiple parameters that could undoubtedly improve sensitivity and specificity of SSCD identification. Indeed, several methods have been proposed, such as air-bone gap, wideband tympanometry, vHIT [28,29,30,31,32]. Future studies should assess how the combination of these parameters could improve the diagnostic of SSCD.

## 5. Conclusions

Our results support the superiority of oVEMP as opposed to cVEMP in the identification of SSCD. oVEMP (4 kHz) positive finding is highly specific for SSCD, but negative findings did not seem to rule out SSCD as the location of dehiscence seems to greatly influence the response rate. Therefore, based on our data, when using VEMP to positively identify SSCD, we recommend applying in the preferred order these cutoff parameters: oVEMP evoked response higher or equal to 10.8 μV, oVEMP threshold lower or equal to 77.5 dBnHL or oVEMP (4 kHz) amplitude of 3.1 μV.

## Figures and Tables

**Table 1:** Description of the different VEMP findings for the different SSCD locations based on CT-scan findings.

			cVEMP		oVEMP	
Localization	n	Age (Years) Mean (±sd)	Peak Amplitude (μV) Mean (±sd)	Threshold (dBnHL) Mean (±sd)	Peak Amplitude (μV) Mean (±sd)	Threshold (dBnHL) Mean (±sd)
Anterior	2	64.5 (±3.53)	584.31 (±168.76)	57.5 (±3.53)	52.24 (±32.55)	60 (±7.07)
Superior	17	58.18 (±10.7)	396.42 (±181.42)	63.53 (±10.71)	40.55 (±24.17)	66.17 (±12.44)
Anterior-Superior	15	50.81 (±168.76)	522.14 (±229.36)	62.81 (±10.94)	60.19 (±47.26)	60.94 (±11.72)
Posterior-Superior	1	30	36.01	95	6.41	95

## Data Availability

The data presented in this study will be made available upon reasonable request to the corresponding author.
